# Boosted Activity of g-C_3_N_4_/UiO-66-NH_2_ Heterostructures for the Photocatalytic Degradation of Contaminants in Water

**DOI:** 10.3390/ijms232112871

**Published:** 2022-10-25

**Authors:** Rafael R. Solís, María Alejandra Quintana, María Ángeles Martín-Lara, Antonio Pérez, Mónica Calero, Mario J. Muñoz-Batista

**Affiliations:** Department of Chemical Engineering, University of Granada, Avda. Fuentenueva, 18071 Granada, Spain

**Keywords:** photocatalysis, graphitic carbon nitride, UiO-66-NH_2_, water treatment, acetaminophen

## Abstract

The combination of graphitic carbon nitride and the metal-organic framework UiO-66-NH_2_ has been developed with the aim to enhance the photocatalytic activity of pure semiconductors. Different proportions of g-C_3_N_4_ and UiO-66-NH_2_ were combined. Complete characterization analysis of the resulting photocatalytic materials was conducted, including N_2_ adsorption isotherms, XRD, FTIR, STEM-EDX microscopy, DRS-UV-visible, and photoluminescence. The photocatalytic activity was tested in an aqueous solution for the removal of acetaminophen as the target pollutant. From the obtained results, less than 50% of UiO-66-NH_2_ incorporated in the g-C_3_N_4_ structure enhanced the photocatalytic degradation rate of both bare semiconductors. Concretely, 75% of g-C_3_N_4_ in the final g-C_3_N_4_/UiO-66-NH_2_ heterostructure led to the best results, i.e., complete acetaminophen elimination initially at 5 mg·L^−1^ in 2 h with a pseudo-first order rate constant of ca. 2 h^−1^. The presence of UiO-66-NH_2_ in the g-C_3_N_4_ enhanced the optoelectronic properties, concretely, the separation of the photo-generated charges was improved according to photoluminescence characterization. The better photo-absorption uptake was also confirmed by the determination of the quantum efficiency values of the heterostructure if compared to either pure g-C_3_N_4_ or UiO-66-NH_2_. This photocatalyst with the best activity was further tested at different pH values, with the best degradation rate at a pH close to the pH_pzc_ ~4.15 of the solid. Sequential recycling tests demonstrated that the heterostructure was stable after five cycles of use, i.e., 15 h. A high contribution of photo-generated holes in the process of the degradation of acetaminophen, followed marginally by superoxide radicals, was suggested by scavenger tests.

## 1. Introduction

Freshwater is one of the most important resources on Earth, which has been threatened in recent decades by anthropogenic activities, raising the need for the removal of pollutants to potentiate the reuse and the implementation of the circular economy concept [1]. The need to ensure the availability and sustainable management of water and sanitation is included in the sixth sustainable development goal of the United Nations 2030 agenda [2]. The traditional water treatment technologies developed for urban sewage must be updated to accommodate current human lifestyle in which multiple different organic compounds are discharged into wastewater effluents. Commonly, many of these complex pollutants are not easily removed by the conventional biological oxidation processes based on activated sludge [3,4]. These organic anthropogenic compounds have been labeled as contaminants of emerging concern, and have been detected at low concentrations, i.e., at the μg·L^−1^ level in the effluent of urban wastewater treatment plants [4,5]. The nature of these pollutants is diverse and includes pharmaceutical compounds, pesticides, personal care products, detergents, flame retardants, plasticizers, and other industrial chemical compounds [6]. Although the consequences for living beings are unknown in the long term in many cases, some of them have been reported as endocrine disruptors since they can alter the functions of hormones, which results in diverse health effects [7,8].

Chemical processes have been demonstrated as an efficient strategy to remove aqueous pollutants that cannot be addressed by traditional methods [9,10]. Among them, diverse techniques, namely advanced oxidation processes, are based on the generation of hydroxyl radicals, a powerful species capable of unselectively oxidizing organic matter. Photocatalysis is a process respectful of the environment and capable of taking advantage of radiation to transform it into chemical energy. The process consists of the activation of a semiconductor when irradiated with photons of energy larger than their bandgap. Then, the electrons of the valence band can move to the conduction band, producing holes in the valence band. The electron–hole pairs can migrate to the surface of the photocatalyst where some reactive species can be produced. Due to the powerful oxidant capability of these species, including superoxide and hydroxyl radicals or photo-generated holes, the photocatalytic abatement of contaminants of emerging concern has been raised as an efficient technology for water treatment [11,12].

Titanium dioxide has been exploited as a semiconductor for water photocatalytic applications due to its abundance, inexpensiveness, low toxicity, and bandgap energy of approximately 3.2 eV, which allows photo-excitation with radiation up to 387 nm [13]. However, great efforts have been paid to overcome the main limitations of titanium dioxide as a semiconductor, i.e., limited absorption of the solar spectrum in the visible region and the high recombination rate of the photo-generated electron–hole pair [14]. In this sense, many materials with photocatalytic activity have been developed to address these issues. For example, graphitic carbon nitride (g-C_3_N_4_) is a two-dimensional π-conjugated polymeric graphitic-like structure built from the covalent bonds between carbon and nitrogen, which has been recognized as a promising semiconductor for environmental applications [15]. g-C_3_N_4_ displays high chemical and thermal stability with a mild bandgap energy of 2.7 eV, which enables the excitation with radiation up to 460 nm [16]. Metal–Organic Frameworks (MOFs) are porous structures built by metal oxo clusters or secondary building units (SBU) and organic linkers acting as a bridge between the SBU. As a result, a highly porous structure with active redox behavior is produced, which can be used as a potential photocatalyst for water purification [13,17]. MOFs display the advantage of an easy design of certain properties such as the size of the porous cages by selecting appropriate organic ligands, or the tuning of the photocatalytic response by the functionalization of the ligand with certain organic groups. UiO-66 is a Zr-MOF first synthesized at the University of Oslo [18]. The structure is made of Zr_6_O_4_(OH)_4_ octahedron oxo clusters as SBU 12-coordinates with benzenedicarboxylic acid molecules, which act as ligands. The UiO-66 structure is extraordinarily stable in an aqueous solution [19]. Moreover, the bandgap energy of UiO-66 can be tuned by the functionalization of the aromatic ring [20,21], giving the functionalized UiO-66-NH_2_ a promising photocatalytic response and lower bandgap since the amino group acts as the antenna of the visible radiation.

The coupling of two semiconductors to build a heterojunction is one of the most interesting strategies to minimize the individual withdrawals of bare photocatalysts; therefore, enhancing the photocatalytic response. An adequate choice of the relative conduction and valence bands positions of the two selected photocatalysts leads to a selective charge transfer, guiding the electrons to the surface of the desired phase of the composite [22]. Thus, the combination of graphitic carbon nitride with other semiconductors, such as TiO_2_ and/or Ag particles, has been proven efficient [23]. The heterojunctions based on UiO-66-NH_2_ have been also reported as a proficient approach [24]. One-pot synthesis routes, when possible, are preferred for promoting good hetero-interfacial contact between the two semiconductors [25]. However, the synthesis of the heterojunction is not always possible in only one step. This is the case for the g-C_3_N_4_/UiO-66-NH_2_ composite. g-C_3_N_4_ is prepared from the thermal decomposition of nitrogen-rich organic precursors under a controlled atmosphere at temperatures as high as 500 °C [16], which is incompatible with the hydrothermal synthesis of MOFs under mild conditions [17]. For that reason, a two-step route in which the MOF grows onto the surface of a previously synthesized g-C_3_N_4_ is required [26].

This work reports the synthesis, characterization, and photocatalytic activity assessment of the heterostructure that combines g-C_3_N_4_ and UiO-66-NH_2_, intending to overcome the limitations of these two semiconductors and produce a synergistic activity in the removal of aqueous pollutants. Although the g-C_3_N_4_/UiO-66-NH_2_ heterojunction has been demonstrated as effective for CO_2_ [27] and Cr^6+^ reduction [28] or H_2_ production, this work is focused on the characterization, catalytic assessment of acetaminophen removal, and quantification of the absorbed radiation based on intrinsic kinetic parameters of the reaction as the quantum efficiency. For the synthesis, UiO-66-NH_2_ has been grown onto the surface of the g-C_3_N_4_ to ensure good interfacial contact between the two semiconductors. Acetaminophen has been selected as the target pollutant since it is labeled as ubiquitous in the list of pollutants of emerging concern due to its frequent detection and the high concentration that is reported [29,30]. Different ratios of g-C_3_N_4_ to UiO-66-NH_2_ have been considered to establish the optimum relative proportion that minimizes the undesirable recombination effect. The photocatalytic response of the optimum sample has been further assessed at different pH values, the reusability in sequential cycles has been tested, and a photocatalytic activation mechanism has been proposed based on a chemical scavenger study.

## 2. Results and Discussion

### 2.1. Characterization of the g-C_3_N_4_/UiO-66-NH_2_ Heterostructures

**Figure 1:**
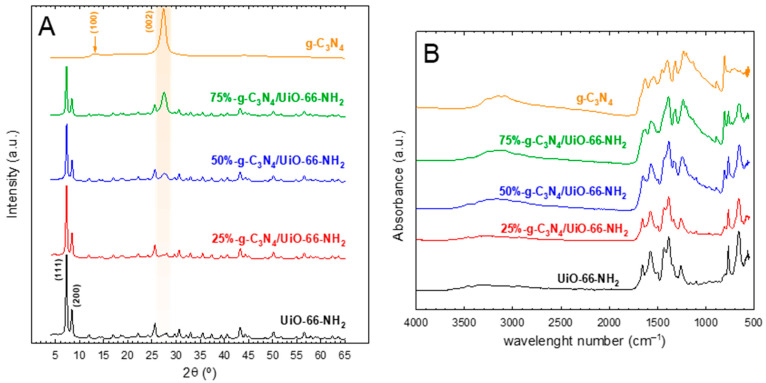
XRD patterns (**A**) and FTIR spectra (**B**) of the g-C_3_N_4_/UiO-66-NH_2_ heterostructures.

The crystalline structure of the heterostructures was assessed by XRD. Figure 1A depicts the XRD diffractograms obtained for pure g-C_3_N_4_, UiO-66-NH_2_, and their combination at different proportions. The patterns obtained for the MOF structure are in good accordance with what is reported in the literature, file CCDC-1405751 [31]. This structure displays two intense peaks located at 2θ = 7.4° and 8.5°, corresponding to (111) and (200) crystallographic planes, respectively. On the other side, the XRD pattern of the graphitic carbon nitride structure generates two peaks [32,33,34]. One is located at 2θ = 13.4°, attributed to the (100) plane, usually at low intensity, corresponding to the heptazine units. In the obtained XRD pattern of g-C_3_N_4_, this peak was poorly defined. However, the second peak that is attributed to the (002) plane, which appears as the consequence of the graphitic character of the interlayered construction, was well-defined. The heterostructures with different g-C_3_N_4_ to UiO-66-NH_2_ ratios displayed the peaks of both crystalline phases, with a clear increase in the (002) peak of g-C_3_N_4_, as the amount of this component was raised. This tendency in the XRD patterns has been observed in the case of this heterostructure [35] or other MOFs, such as NH_2_-MIL-125, when combined with graphitic carbon nitride [26]. 

FTIR analysis was conducted to confirm the formation of the structures of the carbon graphitic nitride and MOF UiO-66-NH_2_. Figure 1B depicts the FTIR spectra of the synthesized heterostructures and pure components. In the case of g-C_3_N_4_, the typical footprint of the vibration of the tri-s-triazine structure was obtained. The vibration of –NH−H and –NH− groups generate a wide band located between 3000 and 3500 cm^−1^. The stretching of the aromatic C−N rings of the tri-s-triazine group produces a group of peaks between 1000 and 1800 cm^−1^. Among them, the most intense peaks, located at 1620 and 1530 cm^−1^ are attributed to the aromatic C−N vibration, while the bands at 1310 and 1230 cm^−1^ are responsible for the stretching vibration of C−N−(C)−C or C−NH−C bonds [36,37]. A very characteristic peak at 810 cm^−1^ is registered in the g-C_3_N_4_ structure due to the interaction of the different layers of triazine rings [38]. The FTIR spectra of UiO-66-NH_2_ displayed some characteristic peaks in response to the vibration of the bonds in the MOF structure, i.e., 2-amino-terephthalic acid (ATA) and the [Zr_6_O_6_(OH)_4_] oxo cluster. At ca. 1660 cm^−1^, a peak appears, representative of the in-plane scissoring bending on N−H_2_ [39]. Two characteristic peaks at ~1390 and 1570 cm^−1^ are attributed to symmetric and asymmetric vibration of the O−C=O bond, respectively [40]. A very low-intensity peak located at approximately 1500 cm^−1^ is representative of C=C aromatic bonds [41]. The bond between the aromatic carbon and the N of the amino group of the ATA molecule generates a peak located at 1280 cm^−1^ [42]. The very intense peak at 765 cm^−1^ is characteristic of the C−H bond [43,44]. Finally, the vibration of the [Zr_6_O_6_(OH)_4_] oxo cluster produces an intense peak at ca. 655 cm^−1^ [21,45]. If the FTIR spectra of pure g-C_3_N_4_ and UiO-66-NH_2_ are compared to the heterostructures containing both, a gradual contribution of the peaks can be observed in each case.

**Figure 2:**
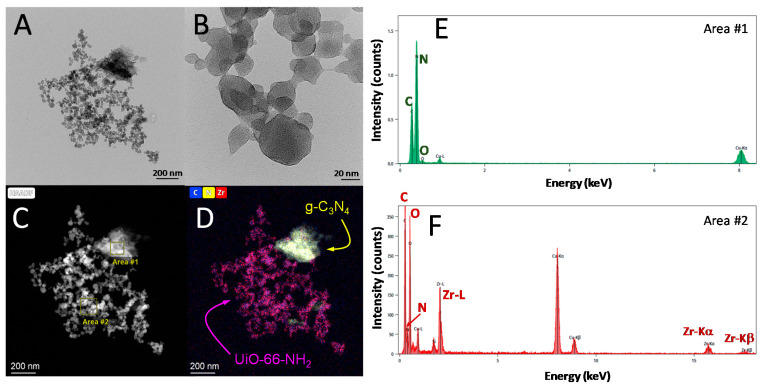
STEM micrographs of the 75%-g-C_3_N_4_/UiO-66-NH_2_ heterostructure. (**A**) STEM picture at 200 nm. (**B**) UiO-66-NH_2_ nanoparticles at 20 nm. (**C**) HAAF image. (**D**) Mapping of C, N, and Zr. (**E**) EDX analysis of g-C_3_N_4_. (**F**) EDX analysis of UiO-66-NH_2_.

The morphological properties were studied by the STEM technique, see Figure 2. From the micrographs obtained, two well-distinguishable phases were observed. The UiO-66-NH_2_ displayed very small particles, ranging from 20–50 nm with an octahedral shape, which were aggregated in bigger groups. The g-C_3_N_4_ appeared joined to some of these aggregations as irregular particles of bigger size, i.e., 200 nm. This heterogeneous interaction has been reported for this heterostructure [27]. An EDX analysis allowed for the mapping of the element composition to ensure the identification of both phases. As depicted in Figure 2, the presence of the carbon nitride region was confirmed as the proportion of nitrogen was higher, see EDX analysis on area #1, Figure 2E. Zr, C, and N at lower intensity were detected in the small particles, a reminder of the octahedral shape as a probe of the UiO-66-NH_2_ presence. An EDX analysis on these particles, area #2, confirmed this hypothesis (Figure 2F). Good contact between the two semiconductors was observed due to the smaller size of the MOF. The UiO-66-NH_2_ particles, ten times smaller, appeared surrounding the g-C_3_N_4_ sheets. However, due to the agglomeration nature of the MOFs particles, not all of them have the same opportunity to be in contact with the g-C_3_N_4_. This aspect predicts that there should be an optimum ratio of UiO-66-NH_2_ proportion, a ratio that promotes the presence of a layer of the MOF with enhanced contact with g-C_3_N_4_. A MOF excess would not necessarily improve the photocatalytic activity since those MOF particles in excess would grow connected to MOF particles onto the already covered g-C_3_N_4_ core. The photocatalytic activity of the samples, discussed in the next subsection, corroborates this theory, proving that the prepared heterostructures with an MOF of less than 50% led to the best results.

N_2_-adsorption isotherms (Table 1) were carried out to assess the textural properties of the synthesized solids. The isotherm of UiO-66-NH_2_ depicted a sharp N_2_ uptake typical of Type I-like isotherms, see Figure S1, with a small hysteresis loop, which confirms the high microporosity and surface area reached in the MOF, specific BET surface area 665 m^2^·g^−1^. This highly developed porosity is in accordance with the literature, in which values up to 900 m^2^·g^−1^ have been reported for the UiO-66 family [46]. In contrast, g-C_3_N_4_ described a Type IV-like isotherm with almost negligible N_2_ adsorption uptake except at high relative pressures, i.e., the BET surface area barely displayed 20 m^2^·g^−1^ and pore volume 0.101 cm^3^·g^−1^. This fact provides evidence of the fairly scarce mesoporosity of a non-porous material. The hybrid heterostructures showed textural properties within the extremes of both phases. Higher porosity was obtained if the relative amount of the MOF was raised. Moreover, the reduction in N_2_ uptake does not necessarily follow a linear tendency with the expected proportion of the MOF in the final solid. This aspect has been attributed to a partial blockage of MOF pores by g-C_3_N_4_ particles [26].

**Figure 3:**
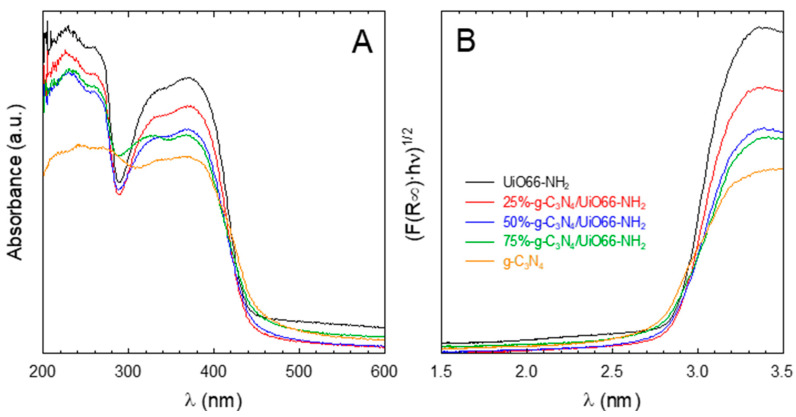
DRS-UV-vis spectra of the g-C_3_N_4_/UiO-66-NH_2_ heterostructures (**A**) and bandgap determination by the Tauc plot method (**B**).

The optical properties were studied by the DRS-UV-visible technique. Figure 3 depicts the absorbance spectra and the estimation of the bandgap values by the Tauc plot method considering them as indirect semiconductors [21]. The absorption spectra of the UiO-66-NH_2_ displayed two well-defined peaks in the UV region. The first one located at ca. 250 nm appears as a consequence of electronic transitions in the secondary built unit of the MOF, i.e., electronic transition from the O atom to Zr in the oxo cluster [47]. A second peak located between 320 and 380 nm is attributed to the ligand-to-metal charge transfer mechanism [48], which means transitions of π electrons from the amino group of the linker to the metal oxo cluster [49]. These two bands in the heterostructures were less defined as g-C_3_N_4_ was incorporated. All the samples extend their radiation harvesting to the visible region, i.e., from 400–420 nm. From the values obtained for the bandgap values (see Table 1), pure UiO-66-NH_2_ presents a higher value than g-C_3_N_4_, i.e., 2.84 vs. 2.70 eV, which is consistent with the already reported values in the literature [16,47]. The bandgap values of the heterostructures obtained by their combination are in accordance with the relative content of both phases, within the limits of the pure phases. 

### 2.2. Photocatalytic Activity of the g-C_3_N_4_/UiO-66-NH_2_ Heterostructures

**Figure 4:**
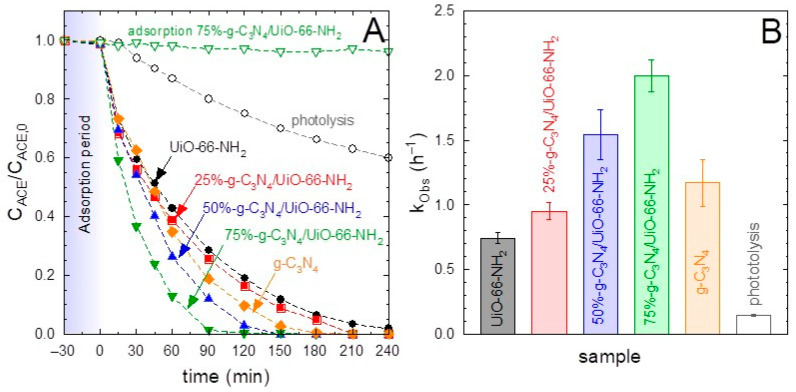
Photocatalytic degradation of acetaminophen (ACE) with g-C_3_N_4_/UiO-66-NH_2_ heterostructures. (**A**) Temporal evolution of acetaminophen with the different samples. (**B**) Pseudo-first order rate constant (k_Obs_) of the different samples. Experimental conditions: V = 350 mL; T = 20 °C, C_ACE_ = 5 mg·L^−1^; C_CAT_ = 0.5 g·L^−1^.

The photocatalytic activity of the g-C_3_N_4_/UiO-66-NH_2_ heterostructures at different relative proportions was tested in the degradation of a target pollutant of emerging concern as acetaminophen (ACE), whose presence in wastewater effluents has been frequently reported [5,50]. Figure 4A shows the results obtained. A negligible contribution of adsorption was observed in all the heterostructures, including the pure g-C_3_N_4_ and UiO-66-NH_2_, during the 30 min adsorption step carried out before irradiation. The sole contribution of 365 nm radiation, i.e., photolysis, led to 40% of ACE degradation in 4 h. The photocatalytic tests led to the complete removal of ACE in all the samples tested after 4 h of treatment. The pure UiO-66-NH_2_ sample displayed less photocatalytic activity than graphitic carbon nitride. If the pseudo-first order rate constants (k_Obs_) are calculated as a mere comparative tool, the g-C_3_N_4_ sample performed ~1.6 times higher value if compared to the MOF, i.e., k_Obs_ (g-C_3_N_4_) = 1.17 h^−1^ vs. k_Obs_ (UiO-66-NH_2_) = 0.74 h^−1^. Regarding the heterostructures, it was observed that the combination of both semiconductors enhanced the photocatalytic activity, reaching improved results if the UiO-66-NH_2_ was minor in the final heterojunction, e.g., less than 50%. According to the pseudo-first order rate constant calculated in each case (Figure 4B), the values followed the order 75%-g-C_3_N_4_/UiO-66-NH_2_ > 50%-g-C_3_N_4_/UiO-66-NH_2_ > g-C_3_N_4_ > 25%-g-C_3_N_4_/UiO-66-NH_2_ > UiO-66-NH_2_. The samples containing 50% and 75% of g-C_3_N_4_ led to a synergistic effect with boosted degradation, which cannot be explained as the relative contribution of the pure phases. Concretely, the sample 75%-g-C_3_N_4_/UiO-66-NH_2_ displayed a k_Obs_ = 2.0 h^−1^, which means ~1.7 folded value concerning pure g-C_3_N_4_ and ~2.7 times the obtained with the pure MOF. An adsorption blank test was carried out with this optimized sample, leading to no removal of ACE during the whole period tested. 

The outstanding photocatalytic activity of 75%-g-C_3_N_4_/UiO-66-NH_2_ was also corroborated in terms of radiation uptake efficiency. The values of quantum efficiency at the beginning of the photocatalytic reaction, e.g., time zero, shown in Table 2, after the estimation of the local volume rate of photon absorption and the initial reaction rate. The values also followed an outstanding performance of the sample 75%-g-C_3_N_4_/UiO-66-NH_2_, Q_E,0_ = 0.052%. 

**Figure 5:**
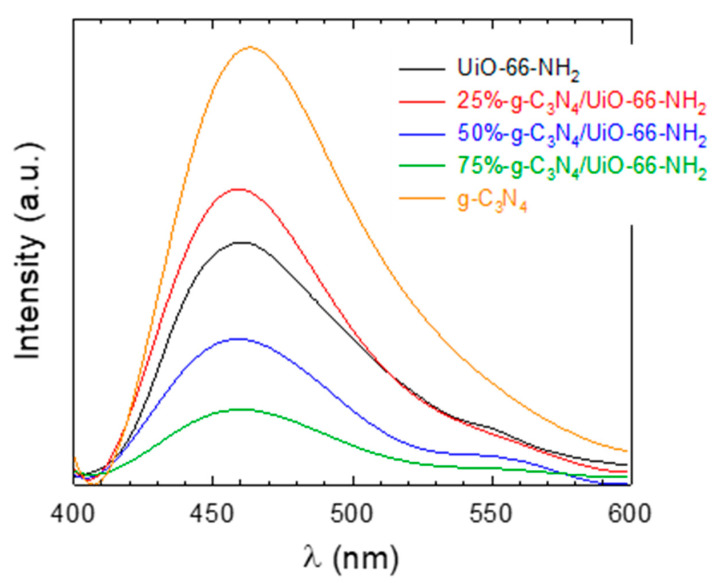
PL spectra of the g-C_3_N_4_/UiO-66-NH_2_ heterostructures.

The optimum activity response of the heterostructures compared to the pure phases cannot be explained according to the bandgap values obtained since the values were higher than the 2.70 eV of pure g-C_3_N_4_ and the radiation used was monochromatic (365 nm). For that purpose, an extra further analysis with the photoluminescence technique was used to assess the effect of the recombination effect of the electron–hole pair, as a plausible reason for the differences observed in the photocatalytic activity. The intensity of the PL peak is linked to the rate of the recombination of the photo-generated charges. Thus, the higher the intensity of the PL peak, the higher the recombination effect, which is undesirable for the process [51]. The PL spectra of all the prepared samples are depicted in Figure 5. From this figure, it is observed that the g-C_3_N_4_ displayed a higher PL intensity located at 462 nm compared with UiO-66-NH_2_, whose maximum was moved slightly to a lower wavelength. It can be deduced that the higher recombination rate of g-C_3_N_4_ is probably due to the higher photo-activity of this sample. If the behavior of the heterostructures is analyzed, the optimum ratios of 50 and 75% obtained in the tests of ACE degradation match the lowest intensity of the PL peak. As the sample 75%-g-C_3_N_4_/UiO-66-NH_2_ performed the best results, it was selected as the optimum sample for further study.

### 2.3. Effect of the pH

**Figure 6:**
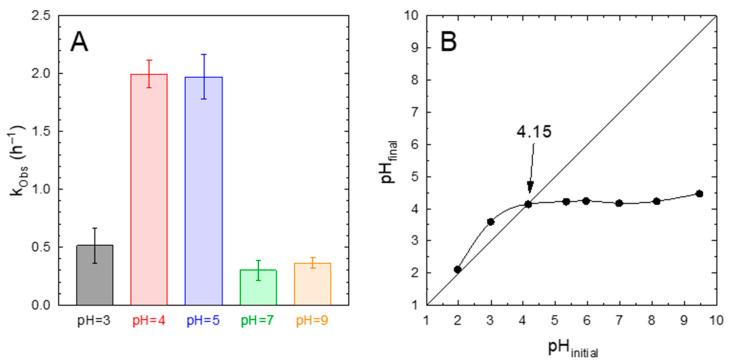
Influence of the pH on the photocatalytic degradation of acetaminophen with 75%-g-C_3_N_4_/UiO-66-NH_2_. (**A**) Pseudo-first order rate constant (k_Obs_). Experimental conditions: V = 350 mL; T = 20 °C, C_ACE_ = 5 mg·L^−1^; C_CAT_ = 0.5 g·L^−1^. (**B**) Determination of the pH_pzc_ of 75%-g-C_3_N_4_/UiO-66-NH_2_ sample by the drift method.

The effect of the solution pH on the photocatalytic performance of the 75%-g-C_3_N_4_/UiO-66-NH_2_ sample was assessed. Figure 6A depicts the influence of pH on the pseudo-first order rate constant. This figure shows an important influence of the pH on k_Obs_. At pH values between 4 and 5, the highest value of k_Obs_ ~2 h^−1^ was obtained. If the pH was raised or decreased from this interval, a drastic drop in the kinetic constant was registered. 

The determination of the pH of zero charge was carried out to explain this behavior. Figure 6B depicts the determination of the pH_pzc_ by the drift method. A value of pH_pzc_ = 4.15 was estimated, which means that the surface of the photocatalyst was positively charged below this value and negatively charged over the pH_pzc_. The values reported in the literature for either UiO-66-NH_2_ or g-C_3_N_4_ are close to that obtained in this work for the heterostructure. A high acidic character of the MOF UiO-66-NH_2_ has been reported, with a value for the pH_pzc_ of 4.3 [52] or even lower, i.e., 3.9 [47]. This acidic character has been ascribed to the change in the protonation stage of the Zr oxo cluster rather than the presence of the amino group [53]. In the case of g-C_3_N_4_, whose presence is a majority in the 75%-g-C_3_N_4_/UiO-66-NH_2_ sample, the pH_pzc_ has been reported at approximately 4.2 [54].

The acetaminophen molecule displays a weak acid behavior, with a pK_a_ = 9.6–9.8 [55,56]. Over the pK_a_, the –OH group is deprotonated, leading to the ACE^−^ anion. However, in the pH range studied during the photocatalytic degradation tests, the acetaminophen molecule was present in the neutral form (ACE^0^); thus, the influence of electrostatic interaction between the ACE^0^ molecule and the surface of the photocatalyst seems to have a low impact on the adsorption step required for approaching the surface where the reactions preferably take place. Other interactions described for ACE with the solid include π-π and hydrogen bonds [57], which would explain why the optimum results reached pH = 4–5, i.e., around the circumneutral conditions for surface charge (pH_pzc_). 

### 2.4. Stability and Reusability

**Figure 7:**
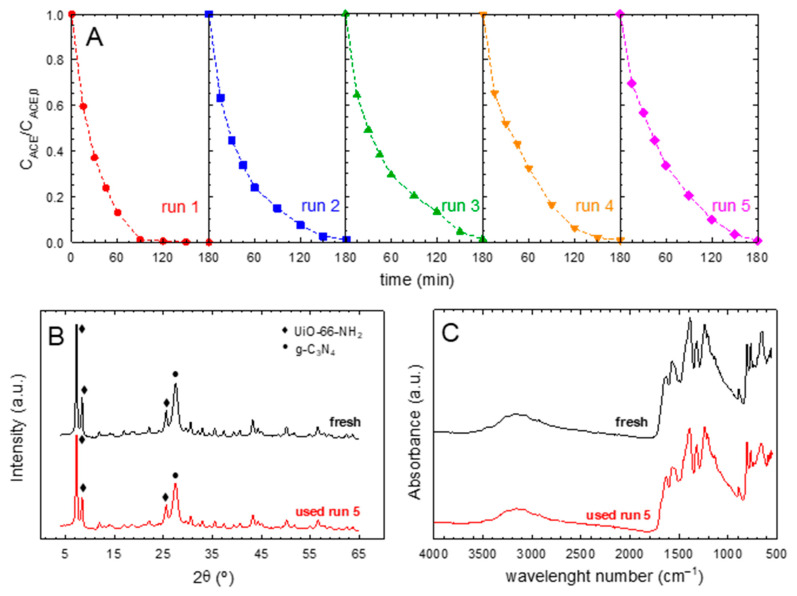
Stability tests of 75%-g-C_3_N_4_/UiO-66-NH_2_ sample. (**A**) Photocatalytic degradation of acetaminophen in sequential experiments of photocatalyst reuse. Experimental conditions: V = 350 mL; T = 20 °C, C_ACE_ = 5 mg·L^−1^; C_CAT_ = 0.5 g·L^−1^. (**B**) XRD diffractograms before and after use in the 5th run. (**C**) FTIR spectra before and after use in the 5th run.

The sample with the optimum ratio of graphitic carbon nitride and the MOF, i.e., 75%-g-C_3_N_4_-UiO-66-NH_2_, was tested in sequential tests of recycling and reusing. Figure 7A shows the results obtained after five consecutive runs. In terms of conversion, it is observed that no significant loss was observed, with acetaminophen being completely removed after 180 min. The kinetics of the process followed a slight decrease in the calculated pseudo-first order rate constant; i.e., 1.90 h^−1^ (1st run), 1.45 h^−1^ (2nd run), 1.42 h^−1^ (3rd run), 1.31 h^−1^ (4th run), and 1.26 h^−1^ (5th run). This marginal decrease in the kinetics parameter could be due to the adsorption of final oxidation products onto the surface of the photocatalyst, subtracting photocatalytic active points for oxidation of new acetaminophen molecules. The stability of the solid was also assessed by analyzing the structure after the final run. As depicted in Figure 7B, no changes in the XRD pattern were registered. The characteristic points of both phases, g-C_3_N_4_ and UiO-66-NH_2_, were well-defined in the reused sample. Less than 10% loss of intensity in the highest peak was reached in the reused sample. Regarding the FTIR spectra, a very similar pattern was obtained after the fifth cycle, with the most characteristic peaks of the heterostructure well-defined, as in the fresh sample. Concretely, the peak located at 810 cm^−1^, characteristic of the interaction of different layers of triazine rings, did not lose intensity. The aromatic C−N vibration (1620 and 1530 cm^−1^) was also well-defined. The peaks responsible for the UiO-66-NH_2_ structure, such as O−C=O (1390 and 1570 cm^−1^), the in-plane scissoring bending on N−H_2_ (1660 cm^−1^), the C−H in the aromatic ring (765 cm^−1^), or the peak at 655 cm^−1^ that appears due to the presence of the oxo cluster unit, were defined in a very similar intensity and shape similar to the fresh sample. Therefore, it can be concluded that the sample displayed stability in terms of photocatalytic activity and structure after five sequential reusing cycles.

### 2.5. Plausible Mechanism of Photocatalytic Degradation

**Figure 8:**
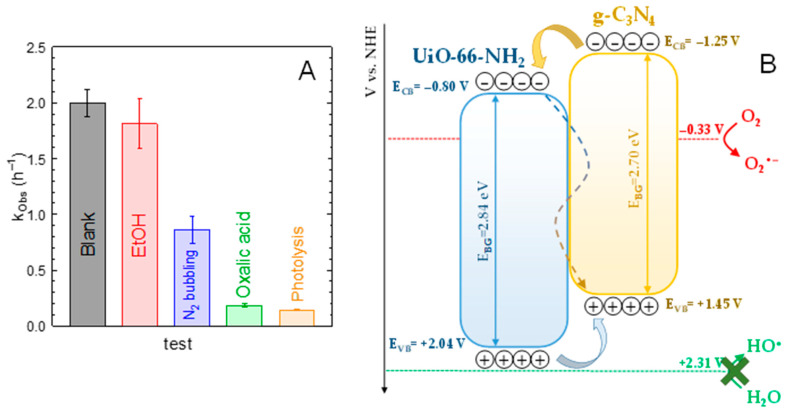
(**A**) Influence of scavengers over 75%-g-C_3_N_4_/UiO-66-NH_2_ sample on the pseudo-first order rate constant. Experimental conditions: V = 350 mL; T = 20 °C, C_ACE_ = 5 mg·L^−1^; C_scavenger_ = 10 mM; C_CAT_ = 0.5 g·L^−1^. (**B**) Bandgap alignment proposal of g-C_3_N_4_/UiO-66-NH_2_ heterostructure.

The influence of the reactive oxidation species (ROS) involved in the process of the photocatalytic degradation of ACE with the 75%-g-C_3_N_4_/UiO-66-NH_2_ sample was tentatively studied by the addition of specific chemical scavengers. Figure 8A depicts the pseudo-first order rate constant for the blank test and the values registered in the presence of different specific chemical scavengers. The role played by superoxide radicals (O_2_^•−^) was studied by replacing the O_2_ bubbling with N_2_. The k_Obs_ in the presence of N_2_ was reduced ~2.3 times compared to the blank test, which provides evidence of the importance played by O_2_^•−^ in the overall photocatalytic scheme. The hydroxyl radical contribution was evaluated by adding ethanol (EtOH), due to the high potential of alcohols to react with HO^•^ [58]. In this case, ca. 26% of reduction in k_Obs_ with respect to the blank test was recorded. It is important to highlight that alcohols have been reported as good HO^•^ scavengers but their presence can also alter the adsorption mechanism of ACE since they may compete with ACE for the initial adsorption sites, which trigger the photocatalytic reactions. Oxalic acid has been reported as a good inhibitor of photo-generated holes (h^+^) [59,60]. Under the presence of oxalic acid, the photocatalytic performance was negligible and the k_Obs_ obtained was very close to that reached under photolysis, therefore, highlighting the importance of the holes in the photocatalytic process. 

Taking into consideration the results attained in the scavenger tests, a mechanism scheme based on the band alignment of both semiconductors in the heterostructure was proposed. The energy of the conduction band for pure UiO-66-NH_2_ has been reported as nearly −0.80 V vs. NHE [47,61,62]. In the case of g-C_3_N_4_, the conduction band is located at ca. −1.25 V vs. NHE [63,64]. That means that the conduction band of the graphitic carbon nitride is more negative than the MOF. Considering the bandgaps values calculated for the pure phases, the conduction bands were located at +2.84 and 2.70 V vs. NHE for the MOF and g-C_3_N_4_, respectively. The resulting heterojunction follows a Type II scheme [65], displayed in Figure 8A. According to this mechanism, g-C_3_N_4_ as the majority species in the heterojunction, would be photo-activated by the UVA radiation; however, UiO-66-NH_2_ could be also. Photo-generated electrons of g-C_3_N_4_ have the possibility of migration to the conduction band of UiO-66-NH_2_. Additionally, they could be recombined with the holes in g-C_3_N_4_ [28]. This band structure would enhance the spatially effective separation of photo-induced charges [27], minimizing the undesirable recombination effect, as suggested in the PL results. 

Considering this bands scheme, neither UiO-66-NH_2_ nor g-C_3_N_4_ display positive enough valence band energy to generate HO^•^. This is the reason why the contribution of HO^•^ was not as important in the ACE degradation, as the test in the presence of EtOH suggested. However, both holes and O_2_^−^ would participate in the oxidation of ACE, with a higher contribution of the first if compared to the second. According to the literature, in the case of UiO-66-NH_2_, the photocatalytic activity has been attributed to the photo-generated holes and/or superoxide radical [47,52] since the valence band is not positive enough to trigger the formation of HO^•^. In the case of g-C_3_N_4_, the superoxide radical has been reported as the main radical species responsible for aqueous pollutants oxidation [66].

## 3. Materials and Methods

### 3.1. Chemicals and Synthesis of the Photocatalytic Heterostructures

The chemicals were at least analytical grade, purchased from Merck^®^, and used as received. HPLC quality acetonitrile (99.9%) was used as a mobile phase in chromatographic analysis. All the stock solutions were prepared in ultrapure water (18.2 MΩ·cm) from a Direct-Q^®^-UV device (Millipore^®^).

The graphitic carbon nitride, g-C_3_N_4_, was synthesized by pyrolysis of melamine (99%) at 500 °C for 2 h. The resulting yellowish solid was suspended in water and the higher particles that decanted were discharged, collecting the suspended particles that were filtered and dried at 80 °C.

The UiO-66-NH_2_ was obtained by a hydrothermal method in the presence of dimethylformamide (DMF, 99.9%), adapting a recipe from the literature [21,47]. Briefly, 3 mmol of ZrOCl_2_·8H_2_O (98%) and 3 mmol of 2-amino-terephthalic acid (99%) were dissolved in 40 mL of DMF in a Scotch bottle of 100 mL. Next, 20 mL of acetic acid (99.7%) was added. The bottles were sealed and introduced in an oven at 120 °C for 24 h. The as-obtained yellowish solid was recovered by centrifugation (4200 rpm, 5 min) and washed with methanol and water.

The hetero-structures g-C_3_N_4_/UiO-66-NH_2_ were prepared following the same procedure during the synthesis of pure UiO-66-NH_2_. Different ratios were considered by adding a certain amount of g-C_3_N_4_, according to the yield achieved for pure UiO-66-NH_2_. The desired amount of g-C_3_N_4_ was added during the precipitation of the MOF precursors in the DMF solution, therefore, promoting the formation of the metalorganic structure onto the suspended graphitic carbon nitride particles. Different mass percentages of g-C_3_N_4_ (X: 25, 50, and 75%) were tested, labeling the resulting photocatalysts as X-g-C_3_N_4_/UiO-66-NH_2_.

### 3.2. Characterization of the Photocatalytic Heterostructures

The crystalline structure was assessed by the X-ray Diffraction (XRD) technique. A Bruker D8 Discover diffractometer equipped with a PILATUS3R 100K-A detector was used, working with a radiation source of Cu Kα (λ = 1,5406 Å). The signal was registered within a 2θ range from 2–65° (30 s·step^−1^, step = 0.02°). 

The textural and morphological properties were studied by N_2_ adsorption–desorption isotherms at 77 K in a 3P Sync 200 apparatus (3P instruments^©^). The total surface area was determined by the BET method (S_BET_), the micropore area (S_MP_) by the t-plot method, the total pore volume (V_T_) from the adsorbed amount at P/P_0_ = 0.99, and the volume of the micropore (V_MP_) by the t-plot method.

The Fourier Transform InfraRed (FTIR) analysis was used to study the nature of the organic stretching bonds and was carried out in a Perkin–Elmer device model Spectrum65 between wavenumber 400–4000 cm^−1^.

The morphology and distribution of the carbon nitride sheet and UiO-66-NH_2_ particles in the generated heterostructure were studied by Scan Transmission Electron Microscopy (STEM) coupled to High-Angle Annular Dark Field (HAADF) detection and Electron Disperse X-Ray (EDX) analysis in a Thermo Fisher Scientific TALOS F200X device.

The optical properties were analyzed by Diffuse Reflectance Spectroscopy (DRS-UV-visible) in a Varian Cary 5E spectrophotometer. The absorbance and reflectance spectra were recorded and the bandgap values were calculated from the application of the Tauc plot method [67,68]. The photoluminescence (PL) technique was carried out as an indirect analysis of the recombination rate of the electron–hole pair. The analysis was conducted in a Varian Cary Eclipse fluorescence spectrometer, fixing 365 nm as the excitation wavelength.

The pH of the point of zero charge in an aqueous solution (pH_pzc_) was determined by the pH drift method [69]. Basically, 50 mL of solutions containing 0.1 M NaCl (99.7%) were placed in Erlenmeyer flasks and the pH was adjusted to a certain desired value between 2 and 9 by adding diluted solutions of NaOH (98%) and/or HCl (37%). Then, 100 mg of the solid sample was added and kept under stirring at 25 °C for 48 h. The final pH value was then measured. The pH_pzc_ was calculated from the intercept between the plotted final vs. the initial pH with the bisector.

### 3.3. Photocatalytic Degradation Tests

**Figure 9:**
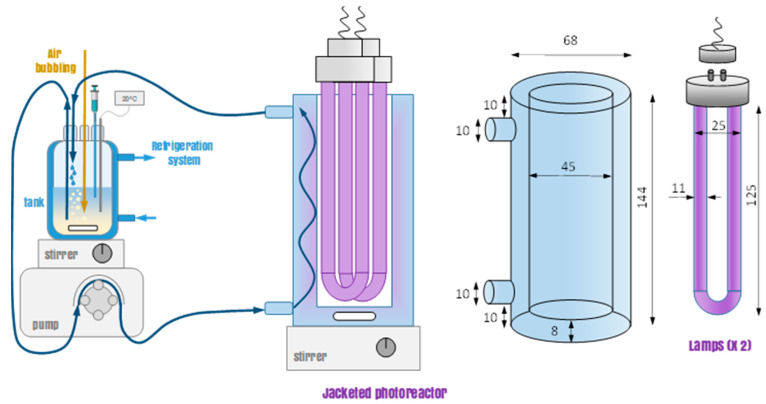
Scheme of the photo-reactor setup (dimensions in mm). Experimental conditions: V_irradiated_ = 200 mL; V_tank_ = 150 mL; T = 20 °C.

The photocatalytic activity of the prepared materials was tested in a UVA photo-reactor equipped with two 9 W lamps emitting at 365 nm. The lamps were placed in the inner space of a jacketed annular reactor made of borosilicate glass, circulating the liquid solution through the jacketing space and magnetically stirred in the bottom. The liquid was pumped and recirculated to an auxiliary tank, also stirred, and equipped with a refrigeration system to maintain the temperature of the solution to 20 °C. Air was bubbled into this tank to keep the solution saturated in O_2_. Figure 9 depicts a scheme of the photo-reactor setup. The irradiating intensity of the lamps was quantified by an in situ chemical actinometry consisting of the photo-reduction of the ferrioxalate complex combined with a polyoxometalate salt (Na_2_SiW_12_O_6_) to register the temporal abatement of the ferrioxalate complex into CO_2_ [70]. The experimental conditions for this determination were oxalic acid (98%) 60 mM, FeCl_3_·6 H_2_O (97%) 5 mM, and H_4_SiW_12_O_40_ (99.9%) 1 mM. The pH of the solution was kept at 4.5 with HCl and NaOH adjustment to prevent the plausible auto-decomposition of the polyoxometalate complex. Taking into account the quantum yield value during the photo-production of (SiW_12_O_40_)^5−^ (ϕ = 0.18 mol·Einstein^−1^ at 365 ± 10 nm) [70], the radiation intensity value estimated was I_0_ = (3.5 ± 0.2) 10^−4^ Einstein L^−1^·min^−1^.

The photocatalytic tests started by loading the aqueous acetaminophen (98%) solution with an initial concentration of 5 mg·L^−1^. Next, the photocatalyst at a dose of 0.5 g·L^−1^ was added to the tank until a homogeneous dispersion was reached. Before irradiation, a 30 min adsorption step was carried out in darkness. After switching the lamps on, samples were extracted from the tank at regular intervals and the photocatalyst was removed with syringe filters (nylon, 0.45 μm). In the experiments carried out in the presence of scavengers, the desired amount of the scavenger (10 mM) was added to the aqueous solution before loading the catalyst. N_2_ was replaced by air in the quenching experiment that assessed the importance of O_2_ (superoxide radical). Oxalic acid was used for suppressing the effect of photo-generated holes and ethanol (96%, vol.) for hydroxyl radicals.

The concentration of the contaminants was quantified by High-Pressure Liquid Chromatography (HPLC) in a Shimadzu LC-10 HPLC device coupled with diode array UV-visible detection. The stationary phase consisted of a Zorbax Bonus-RP column (4.6 × 150 mm, 5 μm). The injection volume was 90 μL. The mobile phase, pumped at 1 mL·min^−1^ under the isocratic mode, was a mixture of 20% acetonitrile (A) and 80% acidified water (B, 0.1% *v/v* H_3_PO_4_), performing the quantification at 247 nm.

**Figure 10:**
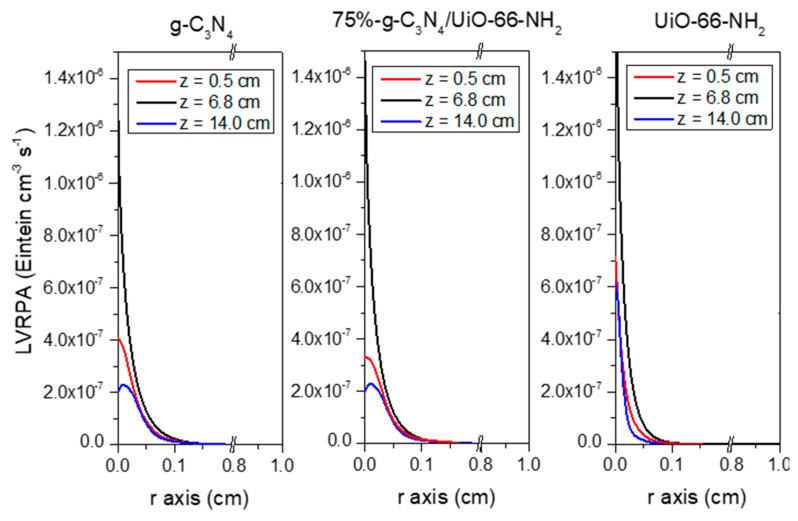
LVRPA profiles of 75%-g-C_3_N_4_/UiO-66-NH_2_ sample and g-C_3_N_4_ and UiO-66-NH_2_ references. The z dimension means the longitudinal distance from the top of the photo-reactor and r the radial position of the jacketed annular space.

The quantum efficiency value (Q_E_) was obtained following the IUPAC recommendations, which establishes the Q_E_ as the ratio of the number of ACE molecules reacting, i.e., the reaction rate, by the number of photons that interact with the photocatalyst, i.e., the local volume rate of photon absorption (LVRPA, e^α,ν^):

(1) $$Q_{E_{0}}=\frac{r_{{\mathrm{ACE}}_{0}}\;\left(\mathrm{mol}\;\mathrm{ACE}\cdot m^{-3}\cdot s^{-1}\right)}{e^{\alpha ,\nu }\left(E\cdot m^{-3}\cdot s^{-1}\right)}$$

where the initial degradation rate (r_ACE,0_) was obtained considering pseudo-first order kinetics, and the photon absorption rate was determined from the radiative transfer equation (RTE), which was solved for the photoreactor from the determination of the optical properties of the photocatalytic suspensions. The followed methodology consisted of a discrete ordinate method in rectangular spectrophotometer cells in combination with a nonlinear, multiparameter regression procedure [71], see Figure S2. The simulation of the photoreactor led to the definition of the photon absorption profiles depicted in Figure 10. The study was carried out for the sample with the best photocatalytic activity as will be discussed in the discussion and results section, i.e., 75%-g-C_3_N_4_-UiO-66-NH_2_. Bare g-C_3_N_4_ and UiO-66-NH_2_ were also considered for comparison purposes. Detailed information about the procedure of the simulation of the LVRPA profiles is described in the Supplementary Information. The LVRPA profiles show that the highest absorption rate was located, as expected, in the center of the z-axis, and gradually decreased with the radial distance. The order among the samples was UiO-66-NH_2_ > 75%-g-C_3_N_4_/UiO-66-NH_2_ > g-C_3_N_4_, which drives the detection of some differences in light penetration through the r coordinate. The relatively high absorption of pristine UiO-66-NH_2_ in comparison with g-C_3_N_4_-containing samples increases the edge absorption effects, which reduces the progressing of radiation in the r-coordinate. Such effect is detected at all the z-positions evaluated. The identification of an absorption profile dominated by the g-C_3_N_4_ structure as well, suggests that the activity enhancement detected must be unequivocally associated with an improvement in the charges photo-handling caused by an optimized g-C_3_N_4_/UiO-66-NH_2_ interface.

## 4. Conclusions

The combination of graphitic carbon nitride with the MOF UiO-66-NH_2_ results in an efficient heterostructure that enhances the photocatalytic activity of the pure phases during the degradation of acetaminophen in water. The optimum proportion suggests a preference for g-C_3_N_4_ as the majority in the final heterojunction, i.e., around 75%, according to the pseudo-first order kinetics and the estimation of the quantum efficiency. This boosted ratio was suggested, according to the characterization of the catalyst, by the lowest recombination effect registered with photoluminescence analysis. STEM pictures combined with EDX mapping confirmed good contact between the two phases, a relevant aspect to promote the electronic transfer between the semiconductors. In addition, as the analysis of the optical properties suggests, the light absorption profile (LVRPA) is dominated by the phase g-C_3_N_4_ of the binary systems, which seems to be in line with obtaining a maximum for the sample 75%-gC_3_N_4_/UiO-66-NH_2_.

The pH during the reaction highly affected the performance of the process and the best results were achieved at a pH close to the pH_pzc_ of the photocatalyst. The solid demonstrated stability and reusability with marginal loss of photocatalytic activity and practically negligible modification of XRD and FTIR patterns. Superoxide radicals, and, especially, photo-generated holes, were the reactive oxidant species responsible for acetaminophen degradation. The bands’ scheme suggests a Type II heterojunction with no contribution to hydroxyl radical formation. 

## Figures and Tables

**Table 1:** Textural and optical properties of the g-C_3_N_4_/UiO-66-NH_2_ heterostructures.

Sample	N_2_ Isotherm				DRS-UV-Vis
	S_BET_ (m^2^·g^−1^)	S_MP_ (m^2^·g^−1^)	V_T_ (cm^3^·g^−1^)	V_MP_ (cm^3^·g^−1^)	E_BG_ (eV)
UiO-66-NH_2_	665	463	1.256	0.250	2.84
25%-g-C_3_N_4_/UiO-66-NH_2_	505	346	0.839	0.177	2.82
50%-g-C_3_N_4_/UiO-66-NH_2_	379	252	0.648	0.130	2.80
75%-g-C_3_N_4_/UiO-66-NH_2_	196	132	0.315	0.065	2.78
g-C_3_N_4_	20	0	0.101	0	2.70

S_BET_: total specific surface area by BET method; S_MP_: micropore surface area by t-plot method; V_T_: total pore volume; V_MP_: micropore volume by t-plot method; E_BG_: bandgap by Tauc plot method.

**Table 2:** Quantum efficiency values of the g-C_3_N_4_/UiO-66-NH_2_ heterostructures.

Sample	e^α,ν^ (Einstein·cm^−3^·s^−1^)	r_ACE,0_ (mmol·cm^−3^·s^−1^)	Q_E,0_ (%)
UiO-66-NH_2_	3.50 × 10^−8^	6.82 × 10^−9^	0.019
25%-g-C_3_N_4_/UiO-66-NH_2_	3.50 × 10^−8^	8.73 × 10^−9^	0.024
50%-g-C_3_N_4_/UiO-66-NH_2_	3.55 × 10^−8^	1.42 × 10^−8^	0.040
75%-g-C_3_N_4_/UiO-66-NH_2_	3.55 × 10^−8^	1.83 × 10^−8^	0.052
g-C_3_N_4_	3.63 × 10^−8^	1.07 × 10^−8^	0.031

## Data Availability

Not applicable.

## Supplementary Materials

The following supporting information can be downloaded at: https://www.mdpi.com/article/10.3390/ijms232112871/s1.
