# Supplementary material for: Boosted Activity of g-C3N4/UiO-66-NH2 Heterostructures for the Photocatalytic Degradation of Contaminants in Water
Source: Int J Mol Sci. 2022 Oct 25;23(21):12871. doi: 10.3390/ijms232112871 (PMC9656334; doi:10.3390/ijms232112871)
Supplement: Supplementary file 1 [file ijms-23-12871-s001.zip › ijms-1956425-supplementary.pdf]

International Journal of
Molecular Sciences

MDPI

Supplementary Information

Boosted Activity of g-C_3N_4/UiO-66-NH_2 Heterostructures for the Photocatalytic Degradation of Contaminants in Water

Rafael R. Solís *, María Alejandra Quintana, María Ángeles Martín-Lara, Antonio Pérez, Mónica Calero and Mario J. Muñoz-Batista *

Department of Chemical Engineering, University of Granada, Avda. Fuentenueva, 18071 Granada, Spain
* Correspondence: rafarsolis@ugr.es (R.R.S.); mariomunoz@ugr.es (M.J.M.-B.)

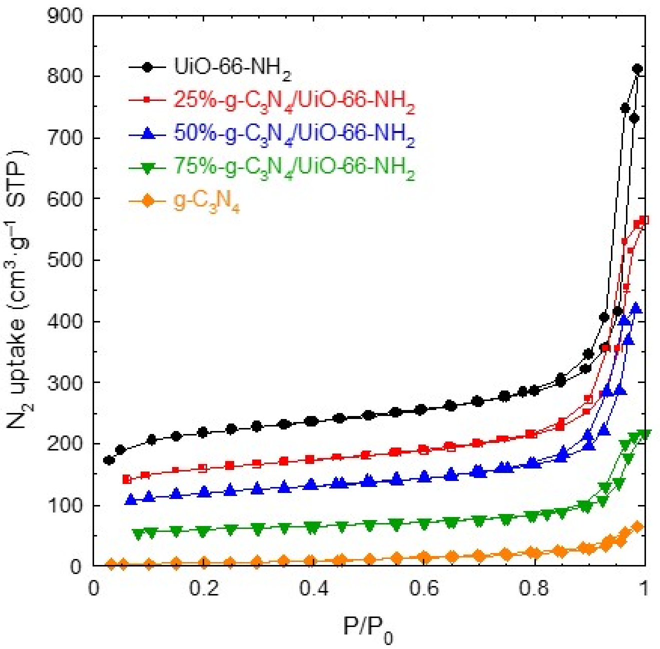

Figure S1. N_2 adsorption-desorption isotherms of the g-C_3N_4/UiO-66-NH_2 heterostructures.

The average volumetric rate of photon absorption was obtained by solving the Radiative Transfer Equation (RTE) in the reactor system schematically presented in **Figure 10** of the main text. The RTE measured the variation of intensity (associated with a beam of rays at wavelength λ in the direction of a solid angle vector, $\underline{\Omega}$) through a direction of the space as described by Eq. S1. The equation assumes (i) the emission radiation is negligible (at room temperature), and (ii) steady state condition during the photocatalytic processes:

$$\frac{dI_{\lambda,\underline{\Omega}}(\underline{x})}{ds} = -\kappa_\lambda(\underline{x})I_{\lambda,\underline{\Omega}}(\underline{x}) - \sigma_\lambda(\underline{x})I_{\lambda,\underline{\Omega}}(\underline{x}) + \frac{\sigma_\lambda(\underline{x})}{4\pi}\int_{\Omega'=4\pi} p(\underline{\Omega}' \to \underline{\Omega})\, I_{\lambda,\underline{\Omega}'}\, d\Omega \quad \text{(S1)}$$

To solve Eq. S1, it is necessary to determine, first, the optical properties of the photocatalytic suspensions. This means the spectral absorption coefficient (κ_λ), the spectral scattering coefficient (σ_λ), and the scattering phase function ($p(\underline{\Omega}' \to \underline{\Omega})$). For that purpose, the extinction coefficient of the samples ($\beta_\lambda = \sigma_\lambda + k_\lambda$) was experimentally obtained by applying a standard linear regression to the plots of β_λ versus catalyst concentration C (6 concentrations from 0.25 - 1.5 g L^{-1}). To obtain the scattering phase function, the Henyey and Greenstein phase function was adopted:

$$p(\underline{\Omega}' \to \underline{\Omega}) = \frac{1-g_\lambda^2}{(1+g_\lambda^2-2g_\lambda^2 u_0)^{3/2}} \quad \text{(S2)}$$

where g_λ is the so-called asymmetry factor and u_0 is the director cosine between incoming and outgoing light at each point of the space.

The optical properties were obtained by solving the RTE using the discrete ordinate method (DOM) in rectangular 0.1-0.5 mm spectrophotometer cells in combination with a nonlinear, multiparameter regression procedure, e.g. Matlab lsqnonlin algorithm: Trust-Region-Reflective Optimization. The cells contain the liquid medium of the reaction and such medium is subjected to agitation in the same conditions used for measuring the reaction rates. Considering that the cell can be represented as an infinite plane parallel medium with azimuthal symmetry, a one-dimensional, one-directional radiation transport model can be used to solve the RTE (see **Figure S2A**). This renders the g_λ, σ_λ, and k_λ values as a function of the wavelength.

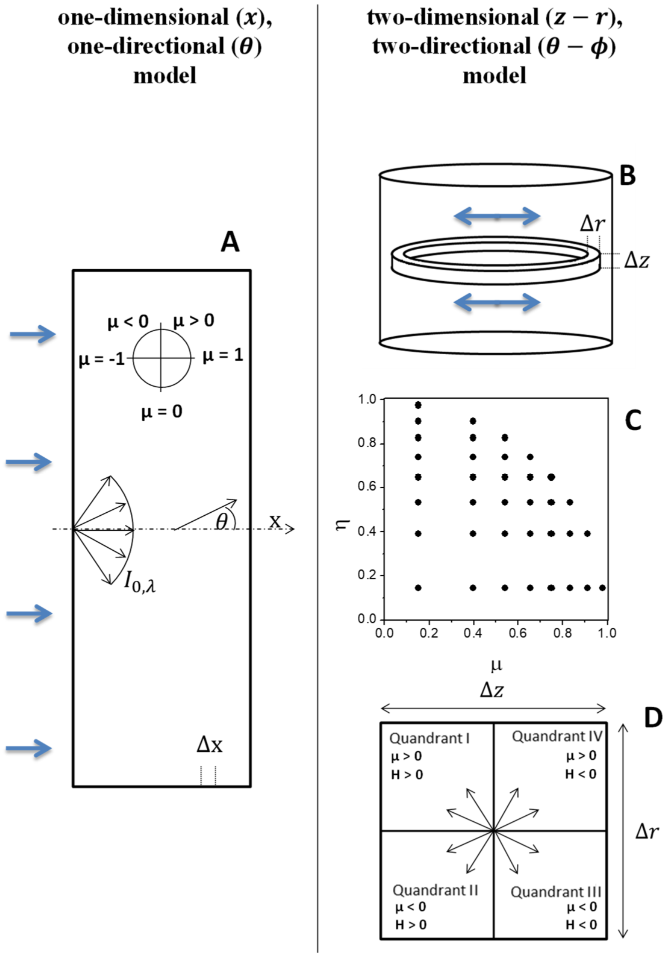

Figure S2. Schematic representation for the one-dimensional, one-directional radiation Model used for the spectrophotometer cell (A) and two-dimensional, two-directional radiation Model used for the photoreactor (B-D). (B) Representation of the r and z spatial mesh discretization of the photoreactor. (C) Directional mesh for the Quadrant I and (D) representation of quadrants of directions as a function of the direction cosines (μ, η) for r and z axis in a cross-section of the spatial cell.

Figure S2 thus shows a schematic representation of the spectrophotometer cell and the corresponding RTE solution scheme where the net light intensity is presented by one angular-related variable (μ=cos θ) at each point of the cell one-dimensional (x variable) representation. The fitting procedure renders the values of ω_λ, the so-called albedo, defined in Eq. S4, and the g_λ parameters that minimize the differences between model predictions and experimental data of diffuse transmittance and reflectance measurements at the spectrophotometric cell for a set of catalyst concentrations, C, and in the wavelength range of the light source. Then, the volumetric scattering and absorption coefficients can be obtained as follows:

$$\sigma_\lambda = \beta_\lambda * \omega_\lambda \tag{S3}$$

$$\kappa_\lambda = \beta_\lambda - \omega_\lambda \tag{S4}$$

Once the optical properties of the samples have been obtained, the evaluation of the radiation field inside the photoreactor can be carried out. The reactor is divided into three zones (defined by the position of the lamp ends) for the calculation. The three zones differ in the boundary conditions as well as the initial position at the radial (r) and axial (z) axis. The DOM tool was used to transform the integral-differential Eq. S1 into a system of algebraic equations that can be solved numerically until convergence with an error below 1 % in each analyzed element. The photo-reactor configuration requires the use of a cylindrical two-dimensional (z-r variables), two-directional (θ-ϕ variables) model of the photo-reactor radiation field (**Figure S2B-C**). The net radiation intensity at each z-r point of the reactor is now represented using a discretized spatial mesh having two angular-related coordinates μ=cos θ and η=cos ϕ. **Figure S2C** displays normalized μ and η values, basal projection(s) in a quadrant of the space around a z-r point calculated using the so-called S_{16} method. The determination of the intensity at each point of the reactor requires dividing the space into 4 quadrants as depicted in **Figure S2D** and the measurement of the incident light intensity at the boundary (liquid surface) using chemical actinometry. According to the Duderstadt and Martin recommendation [1], and following the numerical procedure scheme detailed by previous authors, the finite difference (DOM) was derived directly from the radiation balance at each mesh cell (**Figure S2D**).

Using the DOM approach the central intensity at each spatial cell ($I_m^{i,j}$) can be calculated from the previous ones starting from the appropriate boundary values ($I_m^{i\pm1/2,j}$; $I_m^{i,j\pm1/2}$) as well as one "auxiliary" one coming from the so-called directional mesh ($I_{m-1/2}^{i,j}$) as:

$$I_m^{i,j} = |\mu_m|\left(A_{i,j+1/2} - A_{i,j-1/2}\right)D^{-1}I_m^{i,j\pm1/2} + 2|\eta_m|B_{i,j}D^{-1}I_m^{i\pm1/2,j} + \left(A_{i,j+1/2} - A_{i,j-1/2}\right)\cdot\left(\nu_{m+1/2} + \nu_{m-1/2}\right)D^{-1}W_m^{-1}I^{i,j}_{m-\frac{1}{2}} + \frac{\sigma_\lambda}{4\pi}\sum_{n=1}^{M} I_n^{i,j}P_{nm}W_nV_{i,j}\,D^{-1} \tag{S5}$$

where:

$$D = |\mu_m|\left(A_{i,j+1/2} - A_{i,j-1/2}\right) + 2|\eta_m|B_{i,j} + \left(A_{i,j+1/2} - A_{i,j-1/2}\right)\cdot\left(\nu_{m+1/2} + \nu_{m-1/2}\right)W_m^{-1} + \kappa_\lambda V_{i,j} + \sigma_\lambda V_{i,j} \tag{S6}$$

$$A_{i+1/2,j} = 2\pi r_{i+1/2}\Delta z_j \tag{S7}$$

$$B_{i,j+1/2} = 2\pi\left(\frac{\left(r_{i+1/2}+r_{i-1/2}\right)}{2}\right)\Delta r_i \tag{S8}$$

$$V_{i,j} = 2\pi\left(\frac{\left(r_{i+1/2}+r_{i-1/2}\right)}{2}\right)\Delta z_j\Delta r_i \tag{S9}$$

The computational calculation at each quadrant of **Figure S2D** begins from the corner of the spatial mesh in which two of the four boundary conditions can be applied. In this case, the boundary conditions are: (i) known inlet radiation for quadrants I and II at z=0; (ii) null reflection in the inner reactor wall surfaces. The directional mesh at each spatial cell is always calculated in ascending m values, selecting as m=1 the angular direction more parallel to the z-r plane in which $I_{m-1/2}^{i,j}$ is approximated to zero. With this extra condition and Eq. S11, the recursive Eq. S6 can be applied to the cell closer to the boundary conditions and so forth.

$$\nu_{m+1/2} - \nu_{m-1/2} = -\mu_m W_m \tag{S10}$$

For each quadrant, the complete set of six intensities required for each spatial mesh cell is obtained by applying Eq. S11-13.

$$I_m^{i,j} = \frac{\left(I_m^{i-1/2,j}+I_m^{i+1/2,j}\right)}{2} \tag{S11}$$

$$I_m^{i,j} = \frac{\left(I_m^{i,j-1/2}+I_m^{i,j+1/2}\right)}{2} \tag{S12}$$

$$I_m^{i,j} = \frac{\left(I_{m-1/2}^{i,j} + I_{m+1/2}^{i,j}\right)}{2} \quad \text{(S13)}$$

These equations consider linear intensity profiles inside each spatial mesh cell (an approximation that has increased accuracy as the finite, delta r and z elements go to cero). Finally, once the intensities were obtained, the local volumetric rate of photon absorption (LVRPA, $e^{\alpha,\lambda}$) was calculated at each r-z point of the reactor according to:

$$e^{a,v} = \int_\lambda \kappa_\lambda(\underline{x}) \cdot \int_{\Omega=4\pi} I_{\lambda,\underline{\Omega}}(\underline{x}) d\Omega \, d\lambda \quad \text{(S14)}$$

References

1. Duderstadt, J.J.; Martin, W.R. *Transport Theory*; John Wiley & S: New York, Ny, USA, 1979.
